# Supplementary material for: “Dysregulated not deficit”: A qualitative study on symptomatology of ADHD in young adults
Source: PLoS One. 2023 Oct 12;18(10):e0292721. doi: 10.1371/journal.pone.0292721 (PMC10569543; doi:10.1371/journal.pone.0292721)
Supplement: S1 Appendix — (DOCX) [file pone.0292721.s001.docx]

The purpose is to better understand lived experiences of young adults with ADHD. Helping medical professionals understand what it is like to live with ADHD may allow increased insight into how symptoms impact different parts of people’s lives and allow healthcare professionals better meet the needs of people with ADHD.

Your participation would consist of a one-hour focus group on Zoom with 3-6 other participants. Sample questions include “how have your symptoms changed over time” and “how does ADHD affect your relationships with other people.”

There are some risks associated with this study including a risk of loss of confidentiality. To minimize this risk, don’t share things said during the group with people outside of the group, only use first names, and call from private location. Focus groups will be video and audio recorded and recordings will be kept in an encrypted location and will be deleted after they are transcribed. Transcripts of the focus groups will be deidentified. We will publish this data without your name or identifying information.

There is also a risk of emotional discomfort as the discussion focuses on personal experiences, please only share what you are comfortable with sharing. I am a mandatory reporter, I am required to disclose if you say you are planning on hurting yourself or someone else, although there will not be questions about this.

You will be compensated with a $15 amazon e-gift card after completing the focus group.

Are you okay with us contacting your clinician/prescriber to verify your diagnosis or verifying your prescription on the state monitoring database, if so can you tell me your full name and DOB.

Your responses to the ADHD symptom screening survey you completed before this meeting will be used in the study.

Would you like us to contact you for future studies?

Do you want us to contact you with the results of the study to provide feedback?

You can change mind on participating at any time for any reason.

**ASK QUESTIONS**

I AM AGE 18 OR OLDER. (Verbal for “No” or “Yes”)

I UNDERSTAND THAT I WILL RECEIVE A COPY OF THIS CONSENT FORM FOR MY PERSONAL RECORDS. (Verbal “No” or “Yes”)

I UNDERSTAND THAT THE FOCUS GROUPS WILL BE RECORDED, INCLUDING VIDEOTAPE AND AUDIOTAPE. (Verbal “No” or “Yes”)

I HAVE READ AND UNDERSTAND THE INFORMATION ABOVE AND AGREE TO PARTICIPATE IN THE FOCUS GROUP. (Verbal “No” or “Yes”)

I HAVE READ AND UNDERSTAND THE INFORMATION ABOVE AND AGREE THAT MY ANSWERS TO THE ADULT ADHD SYMPTOM SCREENING CAN BE USED IN THE STUDY (Verbal “No” or “Yes)

I HAVE READ AND UNDERSTAND THE INFORMATION ABOVE AND AGREE THAT I MAY BE CONTACTED TO PARTICIPATE IN FUTURE STUDIES FOR PEOPLE WITH ADHD (Verbal “No” or “Yes)

I HAVE READ AND UNDERSTAND THE INFORMATION ABOVE AND AGREE TO BE CONTACTED WITH THE RESULS OF THIS STUDY TO PROVIDE FEEDBACK. (Verbal “No” or “Yes”)

I HAVE READ AND UNDERSTAND THE INFORMATION ABOVE AND AGREE TO LET THE RESEARCH TEAM CONTACT MY CLINICIAN OR MEDICATION PRESCRIBER TO CONFIRM MY ADHD DIAGNOSIS (Verbal “No” or “Yes)

I HAVE READ AND UNDERSTAND THE INFORMATION ABOVE AND AGREE TO LET THE RESEARCH TEAM VERYIFY MY PERSRIPTION WITH THE PUBLICALLY AVAILABLE CONNECTICUT STATE PERSCRIPTION MONITORING SERVICE (Verbal “No” or “Yes)

I HAVE READ AND UNDERSTAND THE INFORMATION ABOVE AND AGREE TO PARTICIPATE IN THE SURVEY STUDY. (Verbal “No” or “Yes”)
