# Supplementary material for: “Dysregulated not deficit”: A qualitative study on symptomatology of ADHD in young adults
Source: PLoS One. 2023 Oct 12;18(10):e0292721. doi: 10.1371/journal.pone.0292721 (PMC10569543; doi:10.1371/journal.pone.0292721)
Supplement: S2 Appendix — (DOCX) [file pone.0292721.s002.docx]

### CONSENT FOR PARTICIPATION IN A RESEARCH STUDY

**YALE UNIVERSITY**

**Study Title:**

A qualitative study of features of ADHD in young adults

**Principal Investigators:** Marc Potenza, MD, PhD

**Research Study Summary:**

- We are asking you to join a research study.
- The purpose of this study is to better understand the lived experiences of adults with ADHD.
- Study activities will include 1) attending an online focus group, where you will be asked about your experiences with ADHD 2) completing a brief online survey that will ask you basic demographic questions (e.g., age, gender, race), questions about your psychiatric history.
- **Please note that the online focus groups will be recorded (video/audio) so that they can be transcribed (typed out into a written document). Video recording will be used to help keep track of which participant is speaking and all video/audio recordings will be destroyed within six months of recording.**
- Your involvement will require approximately 70 minutes (60 minutes for the focus group; 10 minutes for the survey).
- We will keep your information confidential. We ask that all focus group members not repeat any information shared during the focus group to others. However, we have no control over what happens outside of the group. Therefore, please, be aware of what you share in the group and do not share anything you hear from others outside of the group.
- The study may have no direct benefits to you, but the results will benefit science and others through increasing our knowledge about how to form diagnostic criteria for ADHD.
- Taking part in this study is your choice. You can choose to take part, or you can choose not to take part in this study. You also can change your mind at any time. Whatever choice you make will not have any effect on your relationship with the study staff, doctors, or Yale University.
- Ask the study staff questions about anything you do not understand before deciding whether to participate (contact information is included at the end of this form).
- Once you understand the study, we will ask you if you wish to participate. If you do not want to participate, click “no” at the end of this form. If you would like to participate, check “yes” at the end of this form and provide your contact information. We only will use your contact information to reach you for scheduling the focus group and compensating you for participating.

**Why is this study being offered to me?**

We are asking you to take part in a research study because you told us that you are between ages 18-35 and have been diagnosed with ADHD by a clinician. You can participate in the study regardless of the age at which you were diagnosed and whether you take medications for ADHD. We are looking for up to 80 participants to be part of this research study.

**Who is paying for the study?**

The Yale School of Medicine Office of Student Research is paying for this study.

**What is the study about?**

The purpose of this study is to better understand the symptoms of ADHD in adults including emotional symptoms, focusing ability, and how symptoms change over the lifespan. By speaking with people with lived experiences of ADHD we hope to have a better understanding of how to form diagnostic criteria in the future.

**What are you asking me to do and how long will it take?**

If you agree to take part in the study, your participation will involve two activities.

1) You will attend an online focus group hosted via Zoom (a free, secure online videoconference platform). A member of our study staff will lead the meeting and you can expect 3 to 6 other participants to attend the meeting as well. During the focus group, the group leader will remind all participants not to share information discussed in the focus group with others. This is to help protect all participants’ privacy. The group leader will then ask questions and lead a conversation to learn about how you define ADHD and what it is like to live with the condition. For example, you might be asked “How does ADHD affect your relationships with other people?” or “How have your symptoms changed since you were a child?”

2) You also will complete a brief online survey where you will be asked questions about basic demographics (e.g., age, gender, race), and your psychiatric history (age of diagnosis, medications etc).

We expect that the study will take 70 minutes of your time. The focus group will last 60 minutes, and the survey should take 10 minutes or less to complete.

**Are there any risks from participating in this research?**

There are no physical risks associated with participating in the study. However, if you decide to take part in this study, it is possible that you may feel upset or frustrated when discussing your experiences. To help protect against this, you do not have to respond to any question that makes you uncomfortable or that you otherwise do not want to answer.

There also is the possible risk of loss of confidentiality. For example, while we will ask participants not to share any information from the focus group, we cannot guarantee that they will not share information with others. To help protect your privacy, we will ask participants only to refer to themselves using their first names. In addition, given that the focus group will be held online, you are free to participate from a location of your choosing. We encourage you to participate from a private location but depending on the location from which you choose to participate, someone may see you or hear you participating in the focus group.

**How can the study possibly benefit me or others?**

You may not benefit from taking part in this study. However, we hope that our results will add to the knowledge about what it is like to live with ADHD to better inform diagnostic criteria in the future.

**Are there any costs to participation?**

You will not have to pay for taking part in this study. The only cost includes the time you will spend participating in the study.

**Will I be paid for participation?**

You will be paid $15 via e-giftcard (e.g., Amazon) for completing the focus group study. You will be paid after you participate in the focus group. According to the rules of the Internal Revenue Service (IRS), payment for taking part in a study may be considered taxable income.

**How will you keep my data safe and private?**

All of your responses will be held in confidence. Please review the following list to understand the practices we have in place to do our best to maintain your privacy.

- We will keep your name and contact information in a secure location (e.g., an encrypted, password-protected computer) and use it only to contact you for scheduling the focus group and for compensating you for participating.
- We ask that all focus group members only use their first names when referring to themselves during the conversation and not repeat any information shared during the focus group to others. However, we have no control over what happens outside of the group. Therefore, please, be aware of what you share in the group and do not share anything you hear from others outside of the group.
- We will record the online focus groups so that they can be transcribed (typed out into a written document). During transcription, no identifying information like names will be included in the document. Rather, participants will be identified using a code. For example, the information that you provide might be labeled in the written document as being reported by “Participant 1”). Once the focus groups have been transcribed we will delete the recording.
- If you agree to participate, you will be given a participant ID number. You will be asked to enter this number into the brief survey you will take. We will not ask you to enter identifying information like your name into the survey.
- Only the researchers involved in this study and those responsible for research oversight (such as representatives of the Yale University Human Research Protection Program and the Yale University Institutional Review Boards) will have access to any information that could identify you that you provide. We will share your information with others only if you agree to it or when we have to share the information because U.S. or State law requires it. For example, we are required to tell somebody if we learn that you plan to hurt yourself, plan to hurt someone else, or currently are hurting a child or an older person. However, note that we will not ask any questions about these topics.
- Once all identifiable private information like names has been removed from the data, we may share the data will another investigator or use it in future research studies. We will not ask you for any additional permission.
- When we publish the results of the research or talk about it at conferences, we will not use

your name.

- If you agree, we will contact your clinician/medication prescriber to confirm your diagnosis of ADHD and/or verify your prescription on the Connecticut Prescription Monitoring and Reporting System. We will not disclose any of your information in this process. If your clinician reports that you do not have ADHD, your data will be excluded from this study. You do not have to agree to us contacting your clinician or accessing the state database in order to participate.
- You may repeat the adult ADHD symptom screening you completed in the survey, or you may give us permission to use the answers you already provided in the survey. We will not use this information in the study without your consent.

**Future contact with results and for future studies**

If you agree, we can contact you with the results of this study to see if they resonate with your experiences and provide opportunity for you to provide feedback. Agreeing or disagreeing to this will not impact your involvement with this study.

If you agree, we will contact you via phone or email for participation in other studies for adults with ADHD. Not agreeing to future contact will have no impact on your involvement with this study.

**What if I want to refuse or end participation before the study is over?**

Taking part in this study is your choice. You can choose to take part, or you can choose not to take part in this study. You also can change your mind at any time. Whatever choice you make will not have any effect on your relationship with the study investigators or Yale University.

**Who should I contact if I have questions?**

Please feel free to ask about anything you don't understand.

If you have questions or if you have a research-related problem, you can call or email the Principal Investigator Dr Marc Potenza at 203-737-3553 or [marc.potenza@yale.edu](mailto:marc.potenza@yale.edu) or investigator Callie Ginapp at [callie.ginapp@yale.edu](mailto:callie.ginapp@yale.edu).

If you have questions about your rights as a research participant, or you have complaints about this research, you call the Yale Institutional Review Boards at (203) 785-4688 or email [hrpp@yale.edu](mailto:hrpp@yale.edu).

I AM AGE 18 OR OLDER. (Verbal for “No” or “Yes”)

I UNDERSTAND THAT I WILL RECEIVE A COPY OF THIS CONSENT FORM FOR MY PERSONAL RECORDS. (Verbal “No” or “Yes”)

I UNDERSTAND THAT THE FOCUS GROUPS WILL BE RECORDED, INCLUDING VIDEOTAPE AND AUDIOTAPE. (Verbal “No” or “Yes”)

I HAVE READ AND UNDERSTAND THE INFORMATION ABOVE AND AGREE TO PARTICIPATE IN THE FOCUS GROUP. (Verbal “No” or “Yes”)

I HAVE READ AND UNDERSTAND THE INFORMATION ABOVE AND AGREE THAT MY ANSWERS TO THE ADULT ADHD SYMPTOM SCREENING CAN BE USED IN THE STUDY (Verbal “No” or “Yes)

I HAVE READ AND UNDERSTAND THE INFORMATION ABOVE AND AGREE THAT I MAY BE CONTACTED TO PARTICIPATE IN FUTURE STUDIES FOR PEOPLE WITH ADHD (Verbal “No” or “Yes)

I HAVE READ AND UNDERSTAND THE INFORMATION ABOVE AND AGREE TO BE CONTACTED WITH THE RESULS OF THIS STUDY TO PROVIDE FEEDBACK. (Verbal “No” or “Yes”)

I HAVE READ AND UNDERSTAND THE INFORMATION ABOVE AND AGREE TO LET THE RESEARCH TEAM CONTACT MY CLINICIAN OR MEDICATION PRESCRIBER TO CONFIRM MY ADHD DIAGNOSIS (Verbal “No” or “Yes)

I HAVE READ AND UNDERSTAND THE INFORMATION ABOVE AND AGREE TO LET THE RESEARCH TEAM VERYIFY MY PERSRIPTION WITH THE PUBLICALLY AVAILABLE CONNECTICUT STATE PERSCRIPTION MONITORING SERVICE (Verbal “No” or “Yes)

I HAVE READ AND UNDERSTAND THE INFORMATION ABOVE AND AGREE TO PARTICIPATE IN THE SURVEY STUDY. (Verbal “No” or “Yes”)
