# Supplementary material for: “Dysregulated not deficit”: A qualitative study on symptomatology of ADHD in young adults
Source: PLoS One. 2023 Oct 12;18(10):e0292721. doi: 10.1371/journal.pone.0292721 (PMC10569543; doi:10.1371/journal.pone.0292721)
Supplement: S3 Appendix — (DOCX) [file pone.0292721.s003.docx]

**Discussion Guide**

- ADHD is medically described as showing developmentally inappropriate inattention, impulsivity, and hyperactivity. Do you feel like that describes how you conceive of the condition?
- How have your symptoms changed since you were a child?
  - What symptoms (if any) have gone away or have manifested differently, what new symptoms have arisen, and how have you adapted to living with these symptoms?
- Do you experience an overall deficit of attention, or can your attention be influenced by the environment? For example, are there times you can focus extremely well and what circumstances promote that? If so, does such hyperfocusing ever impair your functioning or cause problems?
- How does ADHD affect your emotions? Do you struggle with labile emotions (emotions that fluctuate substantially)? How does ADHD affect your relationships with other people?
  - Do you experience rejection sensitivity dysphoria (feeling particularly sad when feeling socially excluded)? If so, how does it manifest?
