# Supplementary material for: “Dysregulated not deficit”: A qualitative study on symptomatology of ADHD in young adults
Source: PLoS One. 2023 Oct 12;18(10):e0292721. doi: 10.1371/journal.pone.0292721 (PMC10569543; doi:10.1371/journal.pone.0292721)
Supplement: Attachment — Submitted filename: Response to reviewers.docx [file pone.0292721.s005.docx]

1. Please ensure that your manuscript meets PLOS ONE's style requirements, including those for file naming. The PLOS ONE style templates can be found at 
<https://journals.plos.org/plosone/s/file?id=wjVg/PLOSOne_formatting_sample_main_body.pdf> and 
<https://journals.plos.org/plosone/s/file?id=ba62/PLOSOne_formatting_sample_title_authors_affiliations.pdf>

The manuscript has been formatted according to the style requirements.

2. Thank you for stating the following in the Competing Interests section: 
"The authors report no conflicts of interest with the topic of this work. Marc N. Potenza has consulted for and advised Opiant Pharmaceuticals, Idorsia Pharmaceuticals, Baria-Tek, AXA, Game Day Data and the Addiction Policy Forum; has been involved in a patent application with Yale University and Novartis; has received research support from the Mohegan Sun Casino and Connecticut Council on Problem Gambling; has participated in surveys, mailings or telephone consultations related to drug addiction, impulse control disorders or other health topics; and has consulted for law offices and gambling entities on issues related to impulse control or addictive disorders. The other authors report no disclosures."

Please confirm that this does not alter your adherence to all PLOS ONE policies on sharing data and materials, by including the following statement: ""This does not alter our adherence to  PLOS ONE policies on sharing data and materials.” (as detailed online in our guide for authors <http://journals.plos.org/plosone/s/competing-interests>).  If there are restrictions on sharing of data and/or materials, please state these. Please note that we cannot proceed with consideration of your article until this information has been declared.

Please include your updated Competing Interests statement in your cover letter; we will change the online submission form on your behalf.

The statement has been added to the competing interests and the competing interests has been added to the cover letter.

3. Please include your full ethics statement in the ‘Methods’ section of your manuscript file. In your statement, please include the full name of the IRB or ethics committee who approved or waived your study, as well as whether or not you obtained informed written or verbal consent. If consent was waived for your study, please include this information in your statement as well.

The full name of the IRB has been included and it now states we obtained verbal consent from individuals who participated in this study.

4. Please include captions for your Supporting Information files at the end of your manuscript, and update any in-text citations to match accordingly. Please see our Supporting Information guidelines for more information: <http://journals.plos.org/plosone/s/supporting-information>.

The caption for the supporting information is now included in the text.

[Note: HTML markup is below. Please do not edit.]

Reviewers' comments:

Reviewer's Responses to Questions

**Comments to the Author**

1. Is the manuscript technically sound, and do the data support the conclusions?

The manuscript must describe a technically sound piece of scientific research with data that supports the conclusions. Experiments must have been conducted rigorously, with appropriate controls, replication, and sample sizes. The conclusions must be drawn appropriately based on the data presented.

Reviewer #1: Yes

2. Has the statistical analysis been performed appropriately and rigorously?

Reviewer #1: N/A

3. Have the authors made all data underlying the findings in their manuscript fully available?

The [PLOS Data policy](https://nam12.safelinks.protection.outlook.com/?url=http%3A%2F%2Fwww.plosone.org%2Fstatic%2Fpolicies.action%23sharing&data=05%7C01%7Ccallie.ginapp%40yale.edu%7C3c1a22871904426d2b2308dadc9a63ee%7Cdd8cbebb21394df8b4114e3e87abeb5c%7C0%7C0%7C638064853038336182%7CUnknown%7CTWFpbGZsb3d8eyJWIjoiMC4wLjAwMDAiLCJQIjoiV2luMzIiLCJBTiI6Ik1haWwiLCJXVCI6Mn0%3D%7C3000%7C%7C%7C&sdata=zq5W4PLklHQ3qMTTx45S1tkmbgr2uvLvaSbYcBDAWZI%3D&reserved=0) requires authors to make all data underlying the findings described in their manuscript fully available without restriction, with rare exception (please refer to the Data Availability Statement in the manuscript PDF file). The data should be provided as part of the manuscript or its supporting information, or deposited to a public repository. For example, in addition to summary statistics, the data points behind means, medians and variance measures should be available. If there are restrictions on publicly sharing data—e.g. participant privacy or use of data from a third party—those must be specified.

Reviewer #1: Yes

4. Is the manuscript presented in an intelligible fashion and written in standard English?

PLOS ONE does not copyedit accepted manuscripts, so the language in submitted articles must be clear, correct, and unambiguous. Any typographical or grammatical errors should be corrected at revision, so please note any specific errors here.

Reviewer #1: Yes

5. Review Comments to the Author

Please use the space provided to explain your answers to the questions above. You may also include additional comments for the author, including concerns about dual publication, research ethics, or publication ethics. (Please upload your review as an attachment if it exceeds 20,000 characters)

Reviewer #1: This qualitative manuscript aimed to explore young adults perceptions of ADHD diagnostic criteria. The authors found discord between patients perceptions of ADHD and the diagnostic criteria and conclude that the current diagnostic criteria for ADHD may not best represent the symptoms experienced in adults. The article is well written, it was a pleasure to read and adds an interesting and useful addition to the literature. I make further recommendations for the authors to consider below.

Abstract:
- Please add gender of sample
- Please add country of participants. We know that there’s a considerable difference in the perceptions of ADHD in USA vs Europe for example

Response: Participant sex and continent have been added to the abstract.

Introduction
- Well written and covers the relevant literature on ADHD symptoms and how these may present in adults
- I think it would be useful to briefly cover about missed diagnosis in childhood and then how ADHD is diagnosed in adulthood (e.g. briefly cover typical methods used to diagnose adult ADHD) and the impact that a delayed diagnosis may have

Response: It is now covered in the introduction that ADHD is underdiagnosed and there are often substantial delays in diagnosis. A brief overview of methods of diagnosis in adulthood has been added along with the potential impacts of a delayed diagnosis.

- It may also be useful to briefly mention how gender may influence ADHD diagnosis being missed in childhood, for example, the inattentive female may be more likely to be missed. On that note, it is interesting that your sample is predominately female and also did not agree with hyperactivity being a core symptom of ADHD – worthy of discussion

Response: It is now included that females and those with the inattentive subtype are underdiagnosed with ADHD and that our sample is predomionantly female.

Method
- Please state country where participants were recruited/research conducted

Response: It has been stated that this study was based out of the United States. The full list of countries where participants were located has been added.

- It’s a real strength that you asked for confirmation of diagnosis and reaching a cut-off on a symptom scale. Please state why you chose not to exclude those who didn’t provide this and did you explore whether answers differed accordingly.

Response: It is now included in the limitations that diagnosis was only confirmed in 63% of participants. As reasons for not confirming diagnosis were largely participants verbally agreeing to have their provider contacted but then the research team not receiving release-of-information documentation or being unable to establish contact with their providers, data from all participants were included in the study. It was not explored whether answers differed accordingly.

Results
- I would like to have seen some comparison in the results exploring whether there were any differences between males/females and also different ethnicities. We know that these can be very important factors in interpreting ADHD symptoms, one which does require more research, it feels a missed opportunity not to qualitatively explore this in your sample. I appreciate you would not want to make any firm conclusions on this, but a reflection and comparison would add to this paper.

Response: Thank you for bringing up this important point. Differences in number of participants reporting emotional dysregulation and rejection-sensitive dysphoria are now included in the limitations, along with comment that these proportions may not be representative of overall prevalence of these symptoms. As the study was predominantly White, there were not enough participants of other racial and ethnic groups to make informed comparisons between groups. We now note that this is a limitation that should be examined in future studies.

- The quotes were well chosen and reflected the points accurately

Response: Thank you for this comment.

Discussion
- Please reflect on how your findings may reflect the country of the sample – for example, would you expect different findings in Europe and why

Response: A paragraph has been added to the discussion detailing different perspectives on ADHD across cultures, particularly the US and Europe. For example, despite there not being differences in prevalence of ADHD between the US and Europe, there is widespread public perception that ADHD is an American condition and that may influence how participants view diagnostic criteria or conceptualize their symptoms.

- Please reflect on the fact that 93% of your sample are likely to be quite educated/advocates on ADHD (being part of social media groups)

Response: It is now included at the beginning of the discussion that the sample was recruited from online communities for adults with ADHD and are likely to be highly invested in advocating for issues surrounding those with ADHD. We also note the highly educated aspect of the sample and cite the need for study of other populations with other levels of education.

- It is good to see here the discussion between women inattentive and males and hyperactive subtype – please bring this in earlier (intro/results).

Response: The higher prevalence of the inattentive subtype in women has now been added to the introduction.

- In the discussion, is it worth reflecting that perhaps the sample are also not sure what are ADHD characteristics and what are characteristics of other co-morbid disorders or general difficulties that a non-clinical sample of young adults may also experience. For example, it may be common young adults starting work may also experience rejection-sensitive dysphoria. Your sample is predominately female and emotional dysregulation is more prominent in females than males regardless of ADHD. This requires discussion, and if you feel appropriate, the conclusion could be tempered. It is hard to conclude one directional line in a qualitative study, particularly where you did not also interview non-ADHD sample.

Response: It is now further discussed in the limitations that the sample was largely female and that this feature may contribute to the findings of emotional dysregulation and rejection-sensitive dysphoria. Further areas for research including comparing prevalence of these symptoms across demographic features within those with ADHD and in comparison to those without ADHD are now included.
