# Supplementary material for: “Dysregulated not deficit”: A qualitative study on symptomatology of ADHD in young adults
Source: PLoS One. 2023 Oct 12;18(10):e0292721. doi: 10.1371/journal.pone.0292721 (PMC10569543; doi:10.1371/journal.pone.0292721)
Supplement: Attachment — Submitted filename: Response to reviewers.docx [file pone.0292721.s006.docx]

Reviewer #2: This is a qualitative study, investigating the perception of the relevance of the diagnostic criteria for ADHD, through focus groups, using the IPA method to analyse the verbatim of the exchanges in the groups.
Forty-three people, mostly women, self-reporting a diagnosis of ADHD, were recruited from online communities around ADHD.
The results suggest a mismatch in the wording and content of the DSM diagnostic criteria, and highlight that certain notions are prevalent in the participants' perception of symptoms, such as rejection sensitivity dysphoria and hyperfocus.
This study has the interest of highlighting the limits of the diagnostic criteria for ADHD in young adults, but I feel that it does not add much to current knowledge, with a rather low level of evidence.
My main criticism is that these notions (RSD and hyperfocus) are widely discussed in forums, websites and social networks about ADHD, and have been for several years, in the United States and elsewhere, long before this study was conducted. It seems fairly obvious to me that there is a reproduction of a fairly standard discourse, and few new hypotheses obtained.
The authors could have mentioned, or possibly evaluated, the frequency of these data in specialized websites and social groups around ADHD.
It seems to me that this is the main interest of a qualitative study to generate new ideas, and I feel that this study does not really succeed in doing so.
Otherwise, the manuscript is clearly written.

My main criticism is particularly illustrated by the content of the introduction. The authors refer to and argue for the hypotheses they draw from their qualitative work, which is a bit surprising: one gets the impression that they lack the openness to all the hypotheses that the IPA methodology recommends. All in all, I fear that the use of the qualitative IPA method will lead to something rather tautological with this approach.

Thank you for these points. Analyzing content of social media posts was considered when conceptualizing this project. However, it was decided not to take this approach due to concerns about using these data for research purposes. Many platforms for adult ADHD explicitly state that no content should be used for research. Even among platforms that do not comment on research usage, the people posting the content are not doing so under the impression that it will be utilized in this manner. Because of this, it was decided to sample people from online communities where adult ADHD is discussed so that participants could engage in the study fully aware that their words would be used for research. Although topics such as rejection sensitivity dysphoria (RSD) and hyperfocusing may have been described for years on social media, they are largely lacking in the published peer-reviewed literature. This study sought to bridge that gap. Questions on RSD and hyperfocusing were directly asked in the focus groups, but the data analysis took an inductive approach, and many topics emerged from the focus groups that we did not expect to find. These include triggers for RSD, ideas on why young adults with ADHD may experience RSD, alexithymia, time blindness, and irritability upon being interrupted when engaged in hyperfocusing. We have added some of this material to the manuscript.

Other Major points

2/ In the introduction, some points are missing such as general explanations on the qualitative method and its interest in this context, discussion on the genesis of the ADHD criteria in the current classifications.

A sentence generally explaining the qualitative method has been added to the introduction. There is also now more context around the history of ADHD.

3/ Method
I am quite surprised to see an IPA study using focus groups.
I am not aware if it has been done before, and the theoretical and practical limitations and problems of this approach should be further discussed.
In the manuscript, this point is only very briefly addressed: (p. 8, l.185 : “Although IPA traditionally utilizes individual interviews (23), focus groups were employed in this study”)
Why did the IPA method was chosen? I have the impression that the analysis is essentially thematic.

An IPA approach adapted for focus groups was chosen because of the focus on participants’ subjective lived experiences and how people ascribe meaning to these lived experiences. We performed analysis on the individual level before performing wider group analysis, consistent with Love et al’s paper detailing how an IPA approach can be modified for analysis of focus groups. More detail has been added to the data analysis and theoretical framework subsections.

Love, B., Vetere, A., & Davis, P. (2020). Should Interpretative Phenomenological Analysis (IPA) be Used With Focus Groups? Navigating the Bumpy Road of “Iterative Loops,” Idiographic Journeys, and “Phenomenological Bridges”. *International Journal of Qualitative Methods*, *19*, 1609406920921600

I wonder whether using group interviews does not also increase the risk of conformity to a standard discourse.

This has been added to the limitations section.

In addition, it would be useful to mention whether all participants took part in the focus groups, I don't think I have read this information.

It has been clarified under the procedure section of the methods that all 43 participants took part in a focus group.

4/ The interview grid (its final version) should be offered to the reader.

The discussion guide has been attached as an appendix.

5/ I appreciated the discussion of the cultural limitations of the study, but I think that limiting, or at least centering this discussion around the 53 reference is problematic. Being French myself, I re-read the quoted article, and it is truly laughable, so full of grotesque clichés about child-rearing in France. Especially since the prevalence of ADHD is about the same as in many other countries, and the low prescription is mainly due to a dominant psychoanalytical model. The current sharp increase in prescribing in France is simply a sign that this model is gradually becoming outdated.

We regret having initially centered the point around cultural differences in ADHD perceptions around this singular article. Additional citations have been added to communicate a broader view of cultural differences.

Minor points
p.4 : I am not sure that the prevalence of 7% in adults is the more consensual data.

It has been clarified that estimates are between 3%-7%.

Reviewer #3: In their submitted paper, Ginapp and colleagues present data from focus groups involving young adults with a diagnosis or symptoms of ADHD. They recruited a total of 43 subjects from online sources who reported being diagnosed with ADHD by a physician and scoring 23 or higher on the ADHD Self-Report Scale for Adults. All subjects completed a screening questionnaire, and if they met the inclusion criteria, they were asked to participate in video- and audio-recorded focus groups that took place over Zoom with participant consent. The recordings were transcribed verbatim, and the transcripts were analyzed using Interpretative Phenomenological Analysis (IPA). The authors examined how participants perceived the official diagnostic criteria. They found that most participants reported that the diagnostic criteria did not accurately reflect their own experiences with ADHD symptoms. Some study participants suggested that executive dysfunction might better capture their symptoms. In addition, participants reported symptom changes over time in the categories of emotional dysregulation, emotional lability, alexithymia, and rejection-sensitive dysphoria (RSD).

The manuscript is well written and the methodology of the study is sound. The results of this study undoubtedly have clinical implications and may be relevant to refining diagnostic criteria.

My only recommendation is that the authors consider rewording the following sentence, "One participant indicated that their rapid shifts in attention prevented rumination and emotional dysregulation." Please rephrase to: "...that his/her rapid attention shifts...". There are numerous sentences throughout the manuscript with a similar structure.

Thank you so much for your reflections on this manuscript.

The use of the singular “they” was employed to be inclusive of pronouns beyond he and she. This was considered especially prudent as 16% of the sample reported being non-binary. We have now communicated this more clearly in the manuscript.
