# Supplementary material for: “Dysregulated not deficit”: A qualitative study on symptomatology of ADHD in young adults
Source: PLoS One. 2023 Oct 12;18(10):e0292721. doi: 10.1371/journal.pone.0292721 (PMC10569543; doi:10.1371/journal.pone.0292721)
Supplement: Attachment — Submitted filename: Response to Reviewers.docx [file pone.0292721.s007.docx]

Reviewer #2: I have carefully re-read the changes made to the manuscript, and for most of the points raised I have received an appropriate response.

I am grateful to the authors for providing the interview grid. I have participated in the conceptualisation and implementation of several studies using IPA, but I do not consider myself a specialist in this method. I am, however, surprised by this grid, which I would not have validated in its current state for a study in which I would have participated. A large proportion of the questions are closed, and in particular half of the questions opening on a subject. This goes against the general spirit of the IPA and I'm a bit worried about strict compliance with the methodology (in addition to my doubts about group analysis).

Knowing the high level of methodological rigour expected by the journal, I think that a reviewer specialising in IPA should give his opinion.
In the absence of this, my doubts do not allow me to support the publication.

Thank you for raising concerns around the discussion guide. Initial directed questions were asked to gauge agreement or disagreement among the group and to understand whether participants had specific experiences. Subsequently, open-ended follow-up questions were asked to better understand participant experiences. This information has been added to the procedure section of the methods. Additionally, the potential that structuring questions in this way influenced the results has been added as a limitation.

Reviewer #4: I think the authors have responded well to the previous comments. My only comment is around the use of verbal consent and how informed consent can be guaranteed. Please could the authors include the specifics of what participants were informed in the method section and a justification as to why written consent could not be obtained. A copy of the participant information sheet and a copy of the verbal script used, should be submitted as additional information.

Thank you for this comment. Verbal consent was utilized and approved by the IRB due to focus groups taking place virtually. It was seen as unnecessarily burdensome to have participants sign the document remotely and return it. This has been added to the procedure section of the methods. The participant information sheet and script for the consent meeting have been attached as supporting documents.
